# Demonstrating the immunostimulatory and cytokine-augmentation effects of bacterial ghosts on natural killer cells and *Caenorhabditis Elegans*

**DOI:** 10.1002/bit.28619

**Published:** 2023-12-07

**Authors:** Sreeja Narayanan, Adheena Panangattu Baburajan, Mumtaz Muhammad, Andrea Joseph, Praveen Kumar Vemula, Sarita Ganapathy Bhat

**Affiliations:** 1 Department of Biotechnology, Cochin University of Science and Technology, Kochi, Kerala, India; 2 Chemical Biology Approaches for Stem Cells and Therapeutics, Institute for Stem Cell Science and Regenerative Medicine (inStem), Bengaluru, Karnataka, India

**Keywords:** bacterial ghost, *C. elegans*, immuno-modulation, immunotherapy, natural killer cells

## Abstract

The potential of bacteria-based immunotherapy lies in its ability to inherently enhance immune responses. However, the “liveness” of bacteria poses risks of bacterial escape, nonspecific immuno-stimulation, and ethical concerns, limiting their acceptability in immunotherapy. In this scenario, nonliving empty bacterial-cell envelopes, named bacterial ghosts (BGs), have emerged as immuno-stimulants with the potential to side-step the limitations of live bacterial therapies. This study demonstrates the capability of BGs in modulating the functionality of NK-92 cells and *Caenorhabditis elegans* (*C. elegans*), as well as perform as cytokine-therapy adjuvants. BGs were obtained through a pH-driven culture method, and were validated for their structural and chemical integrity via electron microscopy and spectroscopy. In NK-92 cells, BGs have shown significant immuno-stimulation by boosting the gene-expression of perforin, granzyme-B, Fas-L, and interferon-gamma by factors of 3.5-, 1.5-, 12.5-, and 8.6-folds, respectively. Combined BG and IL-12 treatment yielded a notable 10.2-fold increase in interferon-gamma protein expression in 24 h. The BGs also significantly influenced the innate immune response in *C. elegans* through the upregulation of lysozyme genes viz., ilys-3 (8.8-fold) and lys-2 (3.1-fold). Our investigation into the impact of BGs on natural killer cells and *C. elegans* highlights its potential as a valid alternative approach for new-age immunotherapy and cytokine augmentation.

## 1 Introduction

Immunotherapy has emerged as a ground-breaking approach in the field of medical research, harnessing the body's immune system to combat cancer. The origins of modern immunotherapy can be traced back to the pioneering work of William Coley in the nineteenth century (Aranda et al., 2019). By injecting heat-inactivated *Streptococcus* and *Serratia marcescens*, known as Coley's toxins, into malignant tissues, Coley observed the remarkable regression of sarcomas, sparking the idea that bacteria could be utilized as a potential treatment for incurable cancers (Maletzki et al., 2012). This seminal discovery paved the way for significant advancements in the field of immunotherapy worldwide. One such success story is Bacillus Calmette–Guérin (BCG), a live attenuated strain of *Mycobacterium bovis* initially developed as a vaccine for tuberculosis was later Food and Drug Administration (FDA)-approved for the treatment of bladder cancer (Cardillo et al., 2021). BCG exemplifies the successful translation of bacteria-based immunotherapy into clinical practice, underscoring the immense potential of harnessing bacterial interactions with the immune system to combat cancer.

Bacterial-based immunotherapy has garnered considerable attention due to its unique ability to stimulate the immune response and potentially provide effective disease control (Gupta et al., 2021). However, the use of live bacteria in immunotherapy presents several challenges, including the risk of bacterial escape, nonspecific immune activation, and ethical concerns, limiting its widespread acceptance in clinical practice (Nguyen et al., 2021). Recently, a novel and promising alternative to live bacteria has been explored—bacterial ghosts (BGs). These are nonlive entities that are characterized by the lack of cytoplasm and genetic material, however, containing various immunostimulatory patterns on their envelope (Chen et al., 2021; Kudela et al., 2010). Pathogen-associated molecular patterns (PAMPs) such as the peptidoglycan, teichoic acid, lipopolysaccharide, etc which are major constituents of the bacterial cell wall are especially important as they are significant activators of the front-line immune cells like the macrophages, dendritic cells and natural killer (NK) cells (Kumar et al., 2019; Nguyen & Götz, 2016).

The primary aim of this study was to conduct a comprehensive investigation into the potential of BGs as a promising and viable immunomodulatory tool. A novel pH-driven method was developed for preparing structurally robust *Staphylococcus aureus* ghost cells, overcoming the challenges posed by laborious and chemically corrosive methods. We have demonstrated the enhanced biocompatibility of BGs, along with their potent immuno-stimulatory effects on NK cells and the model organism *Caenorhabditis elegans* (*C. elegans*). The synergistic roles of BGs and interleukin-12 (IL-12) in the in vitro priming of NK-92 cells, a clinically applicable cell line, is also verified. Through time-dependent gene expression analyses of critical NK cell markers, viz., perforin, granzyme-B, Fas ligand, and interferon-gamma, we assessed the immuno-priming functionality of BGs and its adjuvant capabilities with IL-12. Additionally, we investigated the effectiveness of BGs as stimulants of the innate immune response in *C. elegans* by assessing the gene expression profiles of lysozyme genes, ilys-3 and lys-2. In total, this study provides valuable insights into the immuno-priming potential of BGs and how they are a promising tool in immunotherapy and cytokine-augmentation therapy, paving the way for further investigations and potential clinical applications.

## 2 Experimental/Methods

### 2.1 Bacterial culture

*S. aureus* (Origin-Microbial Type Culture Collection, India) was used for the preparation of ghost cells. The culturing was done in nutrient broth at 37°C and all the microbial culture consumables were obtained from HiMedia.

### 2.2 Cell culture

NK-92 cell line was purchased from the American Type Culture Collection, and were cultured and expanded in MEM-alpha (Invitrogen) supplemented with 12.5% fetal calf serum (Sigma-Aldrich), 12.5% Horse serum (Himedia), 2 mM glutamine (Sigma-Aldrich), 1 mM sodium pyruvate (Sigma-Aldrich), 1× nonessential amino acids (Sigma-Aldrich), 100 U/mL penicillin (Sigma-Aldrich), 0.1 mg/mL streptomycin (Sigma-Aldrich), 200 U/mL IL-2 (PeproTech), 0.2 mM myoinositol, 0.02 mM folic acid, and 0.1 mM beta-mercaptoethanol.

### 2.3 Preparation of *S. aureus* ghost cells and process scale-up

*S. aureus* cultures at OD = 1 was inoculated in sterile medium conditioned at pH from 2 to 12 at a culture:medium vol/vol ratio of 1:10. The cultures were incubated for overnight (∼12 h), after which 10% EZBlue reagent (HiMedia) was added into the cultures and further incubated for 6 h. The mouth of the culture tubes was wrapped with aluminium foil to avoid prolonged exposure of the reagent to light which may lead to less sensitive results. After incubation, the samples were transferred to microplates and the absorbance was determined at 570 nm, with 600 nm as a reference wavelength, using a Tecan multimode plate reader. The reduction of the dye indicated the extend of metabolically active cells with respect to the control sample at pH 7 (neutral). We also streak-plated the samples obtained after treatment from pH 7–12 and allowed it to incubate at 37°C for 24 h to check potential culture grow-back.

For a scale-up preparation of BGs from *S. aureus*, a 50 mL culture (at OD = 1) was washed and resuspended in sterile culture medium at pH 7 and 12 and allowed to incubate for overnight. After incubation, the metabolic activity of the culture was confirmed as mentioned in the above section. In a parallel experiment, after incubation, the culture was pelletized and washed three times with normal saline by centrifugation at 5000 or 10,000 rpm for 15 min after which the particles were taken for further characterization.

### 2.4 Characterization of the BGs

#### 2.4.1 Scanning electron microscopy (SEM)

The purified BGs were fixed in 1.25% glutaraldehyde for 15 min at room temperature followed by incubation at 4°C overnight. The fixative was then removed completely by repeated washing in double distilled water. This was followed by an alcohol gradient dehydration by incubating the sample in 20%, 40%, 60%, 80%, and 100% ethanol for 10 min each at 4°C. The imaging was done under the scanning electron microscope (JSM 6490 LA, JOEL) to confirm the structural integrity of the BGs.

#### 2.4.2 Gel electrophoresis and Fourier transform infrared (FTIR) spectroscopy

The DNA (from BG as well as from the parent organism) was isolated using the HiPurA Bacterial Genomic DNA Purification Kit (HiMedia) as per manufacturer's instructions. Following the isolation, the DNA content was quantified using a NanoDrop spectrophotometer (Thermo Scientific). Then, the samples were electrophoresed on agarose gel (1.5%) made in TAE buffer (40 mM Tris-acetate, 1 mM ethylenediaminetetraacetic acid) and casted on electrophoretic gel tray after adding ethidium bromide (EtBr). Along with the experimental samples, a suitable DNA size marker (ladder) was also loaded. The gel was run at 50 V for as long as the dye migrated to two-third of the gel distance after which the electrophoresis was stopped and the DNA bands were visualized using a gel documentation system (Syngene G: Box F3, Synoptics Ltd.).

The FTIR spectroscopy (Nicolet™ iS50 FTIR Spectrometer, Thermo Scientific) was done to analyze the chemical integrity of the prepared BGs. The scanning was done for a range of wavenumbers from 4000 to 500 cm^−1^. Freeze-dried samples were evenly mixed with KBr as the reference before the scanning was performed.

### 2.5 Interleukin-12 binding on BGs

Human recombinant IL-12 (Sigma) was bound onto the BGs by co-incubating them in an aqueous suspension of BG at BG:IL12 concentration ratios of 10:1, 20:1, 30:1 for predetermined time intervals. After incubation, the formulation was centrifuged at 5000 rpm for 15 min and the supernatant was collected to quantify the unbound IL12 using human IL-12 sandwich enzyme-linked immunosorbent assay (ELISA) (Sigma) following the manufacturer's protocol.

### 2.6 Quantitative real-time PCR (qPCR)

Total RNA was first isolated using the RNeasy plus mini kit (Qiagen) after treatment of NK-92 cells (2 × 10^6^ cell/well) with BG, IL12, and BG_IL12 for 6, 12, and 24 h. The final concentration of IL12 tested was fixed at 20 ng/mL (the corresponding BG concentration in BG_IL12 was 488.28 ng/mL). The complementary DNA (cDNA) was synthesized using SuperScript III reverse transcriptase kit and random hexamers (Invitrogen) in a total volume of 20 µL. SYBR Green RT-PCR Master Mix (TaKaRa) was used for expression analysis of the gene and the quantification of fold change was done by the 2^−ΔΔCT^ method. All experiments were performed in three individual sets of triplicates. Table 1 represents the the primer set used in this section.

### 2.7 Quantification of protein expression

Interferon-*γ* (IFN*γ*) in the supernatant after sample treatment was quantified using IFN*γ*—specific sandwich ELISA (Invitrogen IFN gamma Human ProQuantum Immunoassay Kit). For this, the NK-92 cells (2 × 10^6^ cells/well) were subjected to treatment similarly as for gene expression study after which, the secreted IFN*γ* in the supernatant were measured following the manufacturer's protocols. All experiments were performed in three individual sets of triplicates.

### 2.8 *C. elegans* experiments

The Bristol N2 strain of *C. elegans* (CGC, University of Minnesota, USA) was maintained at 20°C on solid Nematode Growth Medium (NGM) containing *Escherichia coli* OP50 as a food source. Eggs laid by mature worms were allowed to hatch and the hatchlings (day 1) were cultured on fresh NGM plates at 20°C. L4 larval stage nematodes were collected using M9 buffer and washed twice with 5 mM phosphate buffered saline (PBS) at pH 7.4 to remove OP50. Treatment plates were made by creating a 3–4 cm diameter spread of BG alone (1 × 10^4^ or 2 × 10^4^ units/plate), and BG:OP50 at unit ratios 1:1 and 2:1. The control plate was spread with OP50 alone (1× 10^4^ units/plate). About 100 worms were picked and allowed to incubate in the respective plates for 12 h after which they were washed with PBS and ground under liquid nitrogen. The worm powder was then used to extract total RNA, prepare cDNA, and analyze gene expression following similar protocols as mentioned above. All experiments were performed in three individual sets of triplicates. Table 2 represents the primer set used in this section.

### 2.9 Statistical analysis

Data were analyzed by analysis of variance followed by post hoc Tukey's test using GraphPad Prism, CA. The results were expressed as mean ± SEM, and differences with *p* < 0.05 were considered significant.

## 3 Results and Discussion

### 3.1 Preparation and validation of BG cells

**Figure 1:**
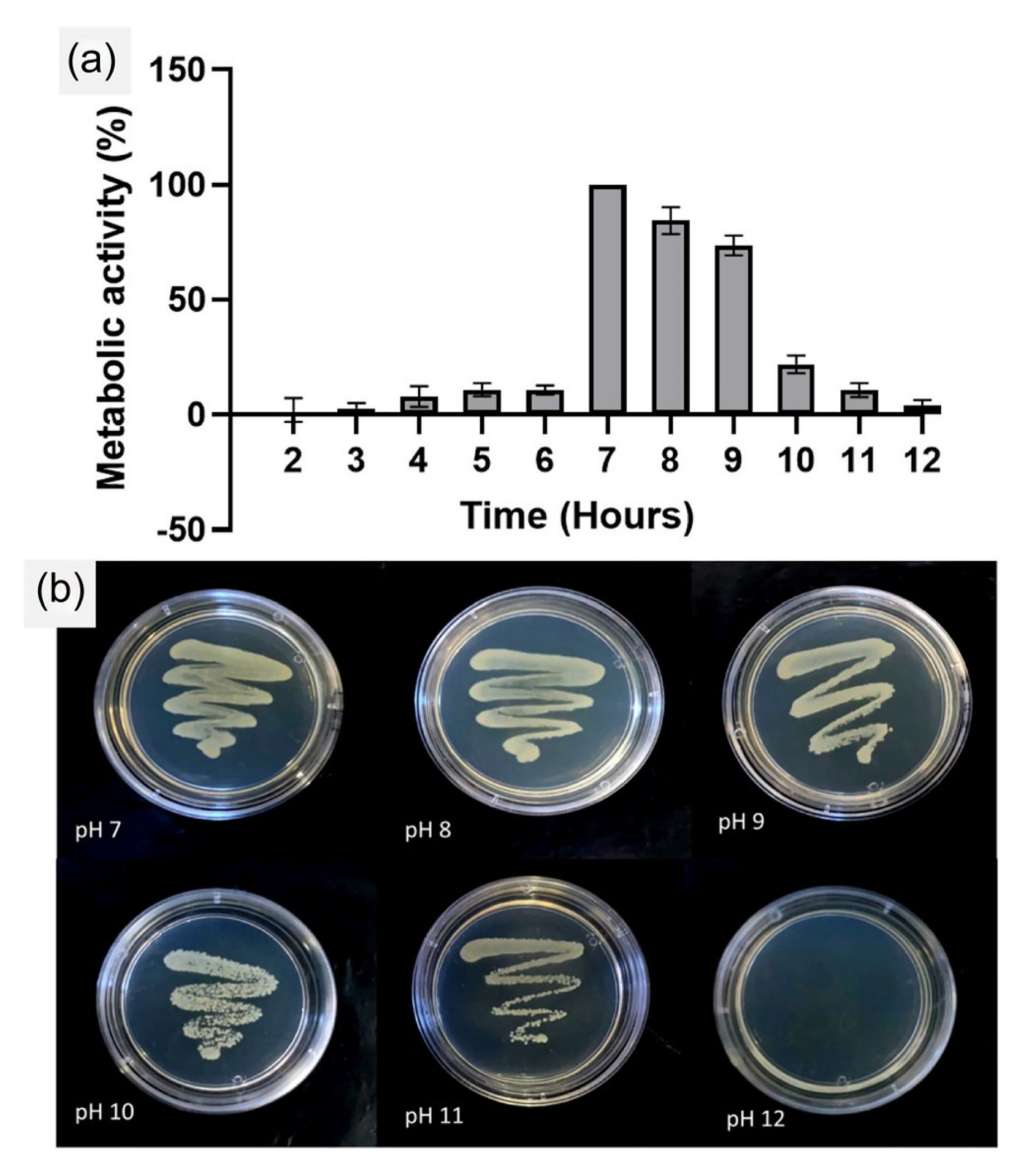
Preparation of bacterial ghost cells. (a) Metabolic activity of *Staphylococcus. aureus* was measured after an overnight culture under various pH conditions, relative to the neutral pH. The graph illustrates the changes in metabolic activity of *S. aureus* at different pH levels, providing insights into the effect of pH on cellular viability and activity during the preparation process of bacterial ghosts. (b) Culture-streaked agar plates depict the revival or non-revival of the *S. aureus* culture after 24 h incubation at pH values ranging from 7 to 12. The images visually demonstrate the ability or inability of the bacterial culture to grow on agar plates after they are subjected to the different pH conditions, indicating the influence of pH in generating ghost cells.

The overall intension of the present study was to develop a bacteria-based ghost cell retaining immunogenic properties that could be utilized as a stimulator for the innate immune system, especially for NK cells. Though researchers have attempted to create BG cells, preparing structurally-integral ghost cells out of Gram-positive bacteria is often limited by the difficulties in perforating their thick outer wall. One limitation with the chemically-driven protocols for BG preparation is about risking the structural integrity of the ghost cell, rendering them excessively perforated, distorted and unusable for biomolecule loading. Maintaining the structural and functional integrity of the ghost cell is an essential prerequisite for using them as drug delivery vehicles, immunotherapy modules and vaccines. Towards this, we prepared structurally sturdy ghost cells of *S. aureus* by culturing them overnight in alkaline culture conditions. From Figure 1a we understand that *S. aureus* cultures when subjected to varying pH conditions (pH 2–12) in their native culture medium succumb to metabolic inactivity, especially at the pH values ≤6.0 and those ≥10.0. The pH 8.0 and 9.0 showed noticeable metabolic activity in comparison to pH 7.0 which was treated as the control (neutral condition) in the experiment. To validate the above results, we plated the treated samples on fresh agar medium. Since, the glycosidic bonds in peptidoglycan (a major component of the BGs) could be sensitive to acid-catalyzed hydrolysis (Bartnik & Facey, 2017), leading to a possible disruption of the cell wall integrity, we decided to work at the alkaline pH range. On observing the plate after 24 h incubation, we found no colonies in pH 12.0, while rest of the pH conditions showed culture revival as in Figure 1b, indicating that *S. aureus* could silently thrive at these pH values (7–11), and revive back when conditions are normal.

**Figure 2:**
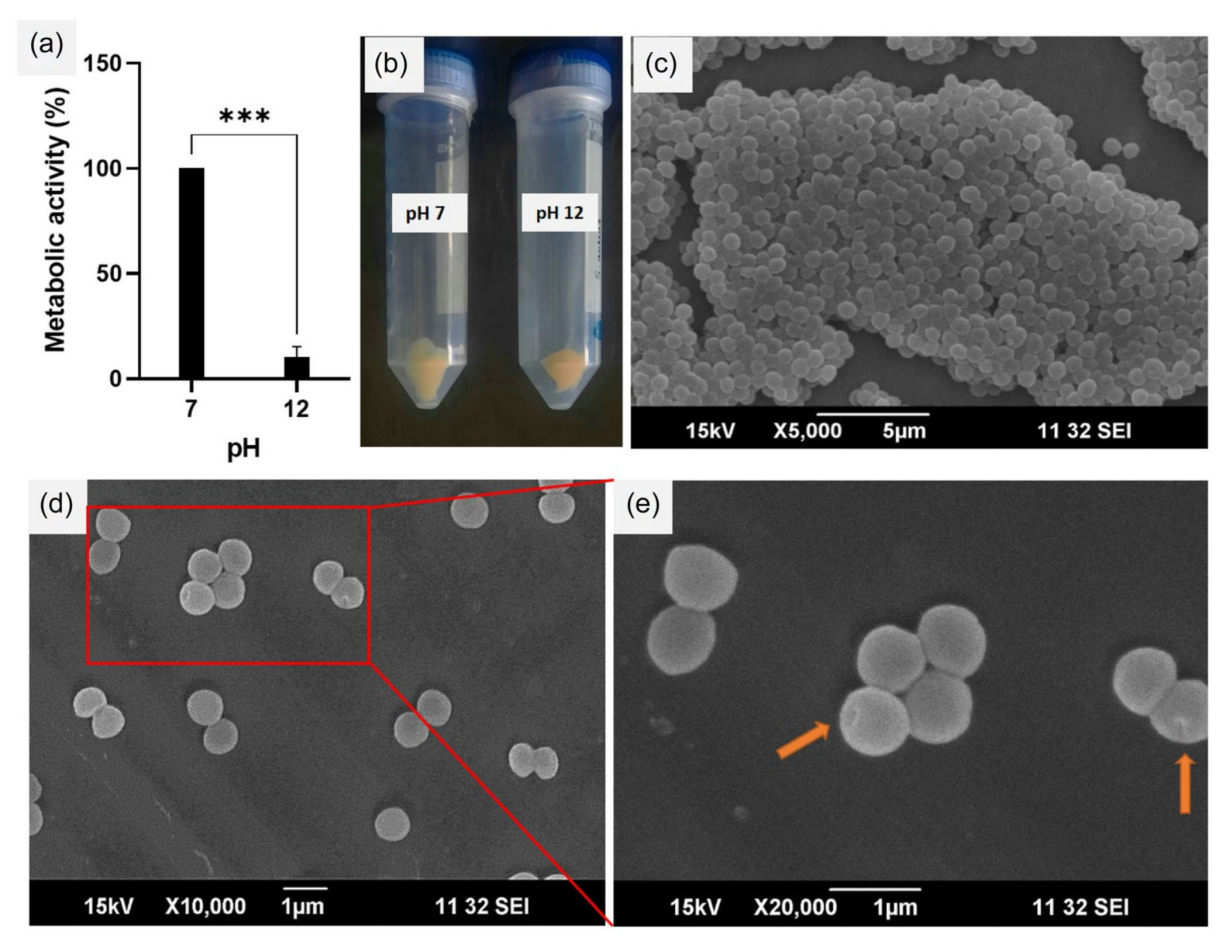
Process scale-up and validation of bacterial ghosts. (a) Metabolic activity assessment of 50 mL *S. aureus* culture at pH 12 and 7, revealing the impact of pH during scale-up (***p < 0.001). (b) Creamy-yellow pellets obtained after centrifugal purification of the scaled-up formulations. (c) Scanning electron micrograph of *S. aureus* cells cultured at pH 7, illustrating normal morphological characteristics. (d) Scanning electron micrograph of *S. aureus* cells cultured at pH 12, showcasing changes as live cells transform into bacterial ghosts. (e) Magnified image showing transmembrane pores on ghost cells obtained at pH 12.

The process scaling-up with a 50 mL *S. aureus* culture maintained at pH 12 also agreed with the above data and shown drastic reduction in metabolic activity to about 10% of that found at pH 7 (Figure 2a). We also determined the dry weigh of the product obtained from the scale-up after centrifugation at 5000 rpm (creamy-yellow pellets, Figure 2b) and freeze-drying. The dry weight of *S. aureus* was confirmed to be 43 ± 4.3 mg while that of the BGs was 25.8 ± 2.1 mg, which was about 56.5% of the dry weight of parent organism. This decrease in weight observed for the BGs could be attributed to the loss of the cellular inclusions viz., the cytoplasm and the nucleus, which constitute a major part of the cellular volume. To examine if an increase in centrifugation speed could yield higher amounts of BGs, we centrifuged the BG suspension at 10,000 rpm and found that even upon increasing the centrifugation speed, the weight of BGs was similar as that obtained at 5000 rpm.

Next, to understand the structural cohesiveness of the ghost cells, we characterized them using SEM (Figure 2c–e). The morphology of *S. aureus* was well-preserved and was ∼1 micron size in diameter, while the BGs showed diameters from 700 to 800 nm, which was slightly smaller compared the parent cells, probably owing to natural shrinkage in size due to the loss of turgidity following the ejection of the cellular inclusions. However, the BGs looked rounded and showed no signs of wrinkles, which was often not the case with previous protocols (Ma et al., 2022; Rabea et al., 2018), possibly due to the distinct differences in the internal and external environment of the bacteria during BG preparation. We also observed trans-membranous pores (around 50–100 nm size) across the bacterial cell wall which according to earlier researchers are the conduits through which the internal components of the cell are expelled leading to hollow ghost structures (Chen et al., 2021; Hajam et al., 2015).

**Figure 3:**
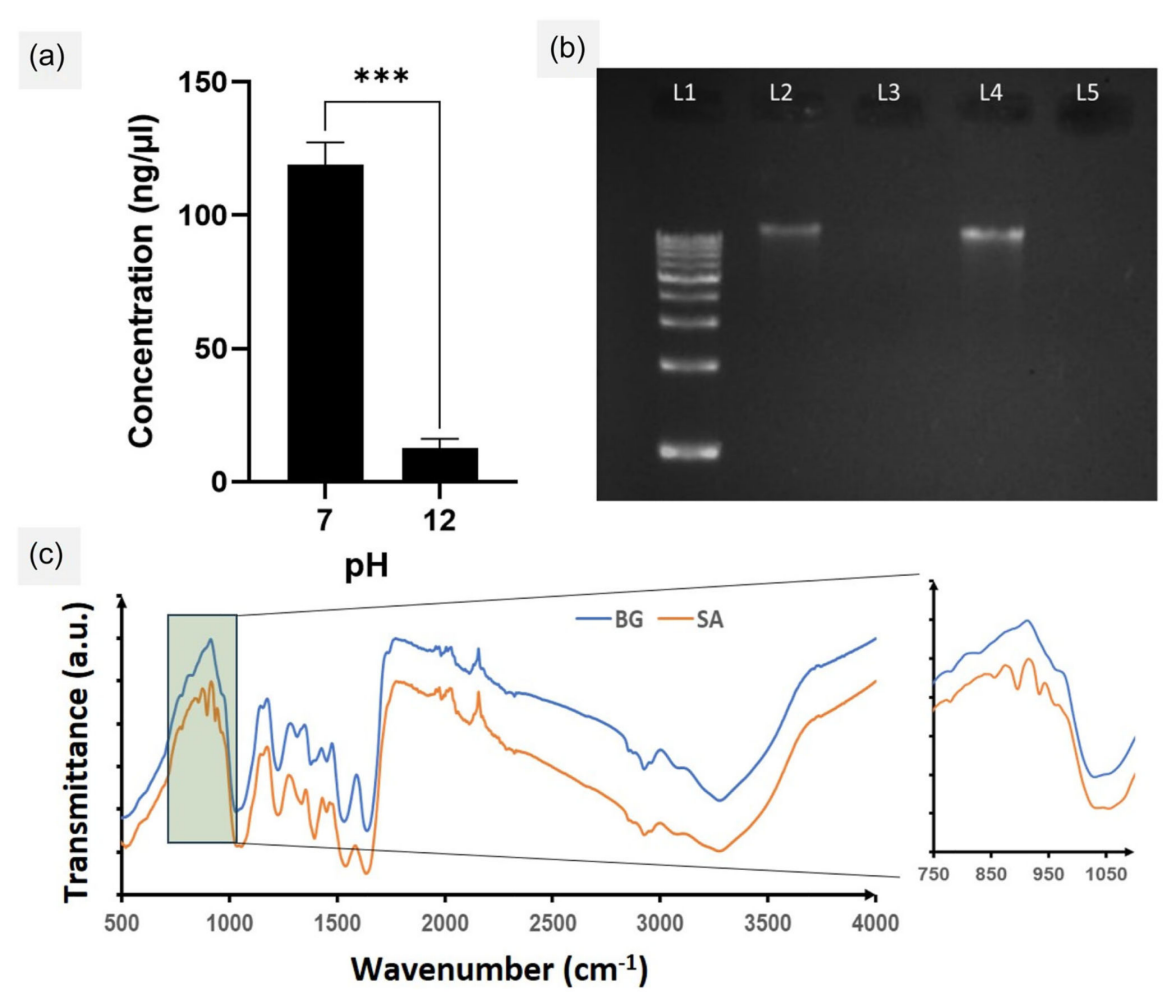
DNA quantification, gel electrophoresis, and Fourier transform infrared (FTIR) spectroscopy (a) DNA derived from *S. aureus* cultured at pH 7 and 12 was quantified using a Nanodrop spectrophotometer to validate the preparation of bacterial ghosts (BGs) (***p < 0.001). (b) Agarose gel electrophoresis (1.5%) was performed using isolated DNA samples from *S. aureus* cultured at pH 7 and 12 for visual comparison. Lanes 2 and 4 represent samples from pH 7, while Lanes 3 and 5 represent samples from pH 12. The loaded DNA sample volumes were 2 µL for Lanes 2 and 3, and 4 µL for Lanes 4 and 5. (c) FTIR spectra obtained for *S. aureus* cultured at pH 7 (SA) and pH 12 (BG). The inset depicts the vibrational bands specific for nucleic acids. BG, bacterial ghost.

To verify the status of DNA in the BGs, we performed the analysis of DNA content in the BGs obtained from a single 50 mL prep using NanoDrop ND 1000 (Thermo Fisher Scientific) and found a quantity of 12.5 ± 3.7 ng/µL isolated from the BGs and 119.1 ± 8.2 ng/µL isolated from cell at pH 7, which was more than 80% higher in content compared to the former (Figure 3a). We then validated the DNA content by electrophoresing the samples on agarose gels. As shown in Figure 3b, intact DNA bands were observed for pH-7 *S. aureus* (Lane 2, 4) as well as for the DNA ladder used as reference (Lane 1). However, no DNA bands were observed for the BGs (Lane 3, 5), indicating that their cores were devoid of any nucleic acid that could effectively be isolated. We also tried increasing the DNA loading volume for the pH-7 sample as well as the BGs and found a thicker band appear for the former only. This was a validation of our BG synthesis protocol, where we had successfully obtained hollow/void structures devoid of nucleic acid.

To examine the status of the PAMPs on the BGs as well as to revalidate the absence of DNA we used the FTIR spectroscopy. The spectrum of BG was compared with that of live *S. aureus* as is shown in the figure (Figure 3c). In both BG and *S. aureus*, the peak at 3272 cm^−1^ typically corresponding to the stretching vibration of the hydroxyl (–OH) functional group was found. This is usually attributed to the carboxylic acid in the D-alanine residue which forms a part of the peptide linker in the peptidoglycan component of bacterial cell wall (Zhang et al., 2023). The sharp peak at 2926 cm^−1^ corresponds to the asymmetric stretching vibration of the methylene (CH2) group in the lipid tails of the plasma membrane (Marseno et al., 2014). Prominent peaks corresponding to the amides from the vibrational stretching band centered around 1635 cm^−1^, amino-acid side chain vibrations was around 1520 cm^−1^ and peaks between 1500 and 1300 cm^−1^ regions due to vibrations of –CH2 and –CH3 from lipids and proteins, were found for both *S. aureus* and BGs with intensity shifts in the latter indicating possible molecular rearrangements in the plasma membrane and the cell wall that could have occurred in the BGs. In general, the conserved biochemical nature of cell wall and membrane of the bacteria which are significant as PAMPs were found unaltered for the BGs as well as the live *S. aureus*, indicating chemical integrity of the prepared BGs (Han et al., 2018; Naumann et al., 1982; Zhang et al., 2016).

However, an interesting aspect was to study the status of sugar-coupled phosphodiester chain vibrations which are seen in the spectral range between 1000 and 800 cm^−1^ (Liquier et al., 1991). Such vibrations are signatures of sugar puckering modes of oligonucleotides and nucleic acids. Our results revealed that bands at 934.17 and 895.28 cm^−1^ corresponding to the vibrations of the sugar-phosphate chain and the deoxy ring in the DNA structure were completely unavailable in the spectrum obtained for BGs, while it was prominently visible in the spectrum for live *S. aureus*. This was in line with previous reports which discussed bands around 950–880 cm^−1^ corresponding to the nucleic acid content of organisms (Zhang et al., 2016). Therefore, our data showing lack of signals form nucleic acids strongly indicated the possible expulsion of the DNA content during the BG preparation process.

**Figure 4:**
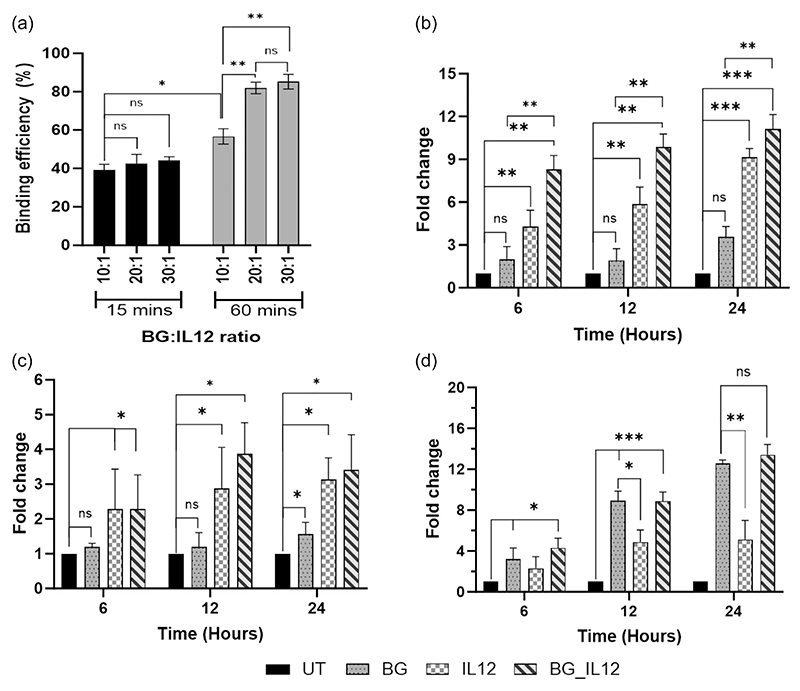
Interleukin-12 binding and gene expression analyses. (a) Quantification of interleukin-12 (IL12) binding with BGs was assessed for varying BG:IL12 ratios and coincubation periods using a sandwich enzyme-linked immunosorbent assay (ELISA). The graph depicts the percentage of IL12 binding, calculated relative to the initial amount of IL12 used in the experiment. Relative quantification of gene expression was performed via qPCR on BG/IL12/BG_IL12-treated NK92 cells using the 2^–ΔΔCt^ method to determine the fold change in the expression of perforin (b), granzyme-B (c), and Fas-L (d), relative to the untreated control. GAPDH was used for normalization. *p < 0.05, **p < 0.005, and ***p < 0.001, ns: not significant. BG, bacterial ghost; GAPDH, glyceraldehyde-3-phosphate dehydrogenase.

Our next attempt was to embed IL-12 over the BGs to investigate synergistic interactions between the two in the activation of NK cells. IL12 is a potent regulator of cell-mediated immune responses and was identified as a “natural killer-stimulating factor” (Kobayashi et al., 1989; Ohs et al., 2017), justifying our choice. Based on our research on previous works highlighting the affinity of cytokines for bacterial cell walls (Mahdavi et al., 2013; Wilson et al., 1998), we devised a co-incubation protocol to investigate this phenomenon further. We first tested a co-incubation of BGs with IL-12 at BG:IL12 weight ratios of 10:1, 20:1, 30:1 for 15 min after which an ELISA was done to quantify the unbound IL12 (Figure 4a). This experiment yielded binding efficiencies (BE) of 39.11 ± 4.61%, 42.36 ± 2.57%, and 44.12 ± 3.89%, respectively, for the tested ratios. Since the BE% obtained was less than half of the initial dose of IL12 used, we decided to increase the coincubation time to 60 min after which a similar analysis followed. After 60 min of coincubation, we found that the BE% had increased to 56.7 ± 3.2%, 81.9 ± 5.7%, and 85.2 ± 2.8% for the ratios of 10:1, 20:1, 30:1. Both 20:1 and 30:1 ratios showed a nearly twofold increase in BE% for 60 min coincubation while 10:1 ratio had about 1.4-fold increase when compared to the previous coincubation time of 15 min. These experiments indicated that by increasing the time of incubation, we could allow more interaction between the BGs and IL12, which was otherwise not possible even upon increasing the BG amounts at a lesser interaction time. Before we embarked on the idea of evaluating the NK cell priming effect of the BGs, we were interested in understanding the compatibility of the BGs on NK-92 cells, which was our in vitro model for subsequent testing. The NK-92 cell line stands as the sole FDA-approved cell line for clinical trials, with numerous immunotherapy demonstrations already done utilizing it (Bergman et al., 2020; Klingemann et al., 2016). We first performed a cytotoxicity assay on NK-92 by subjecting them to differing doses (0–2.5 µg/mL) of BGs and live *S. aureus* (SA) (Figure S1). We found that the cells could tolerate the BGs more than they did the live organism as was evident from our cytotoxicity results. The culture media also eventually turned turbid due to the multiplication of the live organism which later must have contributed to ill-health in the cultures. In contrast, the BG-treated medium remained clear and the cells showed appreciable metabolic activity even after 24 h. Interestingly, the BG data was in strict agreement with another biocompatible particle viz., PLGA nanoparticles, whose assessment was also parallelly conducted as a control experiment as seen in Figure S1. This data indicated that BGs could maintain cytocompatibility like PLGA nanoparticles in the tested dose range.

Our major interest was to investigate a new cell priming method for improvising the functionality and activity of NK-92 NK cells. These *off*-*the*-*shelf* clinically applicable cells are often manipulated by single/combinations of cytokines such as IL-2, 12, 15, 18, 21, and type-1 interferon (Bergman et al., 2020). However, the cost barrier often prevents patients from receiving the benefits of NK-92 priming, limiting their accessibility and practicality in clinical settings. In such a framework, the use of BG cells (a natural collection of discreetly arranged PRR agonists) seems ideal, also because of the affordability in processing them. We intended to do a gene expression profiling for an array of genes coding for the cytolytic markers viz., perforin, granzyme, and apoptosis marker Fas-ligand (Fas-L), as these are important indicators of the cytotoxic functionality of NK cells (Maki et al., 2001). We were also interested to check if the activated NK-92 cells could induce the production of IFN-*γ*, which plays a key role in activating the cellular immunity and consequently, stimulating the antitumor immune-response. A time-dependent treatment of NK-92 cells was done using BG_IL12 particles containing 20 ng/mL of IL-12 (corresponding to 488.28 ng/mL of BGs as per binding efficiency), and was compared with equivalent concentrations of free BG (488.28 ng/mL) and IL-12 (20 ng/mL). Earlier reports on cytokine combination therapy have suggested 20 ng/mL as a sufficient dosage for IL-12 (Guia et al., 2008; Sun et al., 2022). Hence, we decided to follow the same in our study.

The cytotoxic perforin/granzyme-B pathway has been traditionally viewed as a primary mechanism that is used by cytotoxic immune cells to encounter transformed cells (Prager & Watzl, 2019; Trapani & Smyth, 2002). Perforin is a calcium-dependent pore-forming protein that creates pores of 5–20 nm on the plasma membrane of the target cell, to facilitate the entry of granzyme-B for cytolysis. The gene expression obtained for perforin and granzyme-B in IL12-treated NK-92 cells showed consistent upregulation for both genes from early hours of posttreatment and reaching to 8.1-folds for perforin (*p* < 0.001) and 2.4-folds for granzyme-B (*p* < 0.05) by 24 h (Figure 4b,c). On the other hand, the BG-treated cells showed only 2.5-folds (*p* < 0.05) and 0.5-fold increase in perforin and granzyme-B expression respectively after 24 h. However, in line with the performance of IL12, the expression profile obtained for both perforin and granzyme-B in the BG_IL12 particle-treated cells were consistently found upregulated for all tested time points and was significantly higher compared to the untreated control. In general, it was also observed that the expression of perforin was higher than that of granzyme-B (at all time points) for every treatment. The discrepancy in the levels of perforin and granzyme-B production could be attributed to the distinct roles these molecules play. Because perforin is a pore-forming protein and is involved in creating the entry pathway for granzyme-B, its production might need to be comparatively higher to ensure effective delivery of granzyme-B into the target cells.

In the case of Fas-L expression, the scenario was contrasting, where, we found that the BGs by themselves could consistently enhance the expression of Fas-L in a time-dependent manner unlike in the case of perforin and granzyme where they exhibited delayed expression (Figure 4d). In contrast, IL-12 alone, though had induced a 4.1-fold increase in Fas-L (*p* < 0.005) by 24 h, did not match with the upregulation profile shown by BG treatment (*p* < 0.001). Interestingly, the Fas-L expression in the case of IL-12 treatment remained stationary after 12 h without any further alteration with time. Earlier it was reported that, IL-12 modulated the STAT4 pathway which triggered the perforin-granzyme signaling (Prager & Watzl, 2019; Trapani & Smyth, 2002), while inhibited the expression of Fas-L (Wang et al., 2014). Given this background, we believe that, the 15.4-fold increase in Fas-L observed for BG_IL12 treatment could majorly be contributed by the BGs, as it was significantly higher than the expression induced by IL-12 alone. On statistical comparison, we found no significant difference in the Fas-L expression induced by BGs alone and that by the BG_IL12 particles, further supporting our claim on the ability of the BGs in rendering NK-92 competent enough to carry out a FasL-mediated immune surveillance. Earlier reports have suggested cross-talks between PRR-agonists and the Fas/Fas-L system as well as Fas-L overexpression (Wang et al., 2014). These findings suggest that BGs can serve not only as efficient carriers for cytokines but also as effective adjuvants in cytokine therapies, thanks to their inherent expression of PRR-agonists.

**Figure 5:**
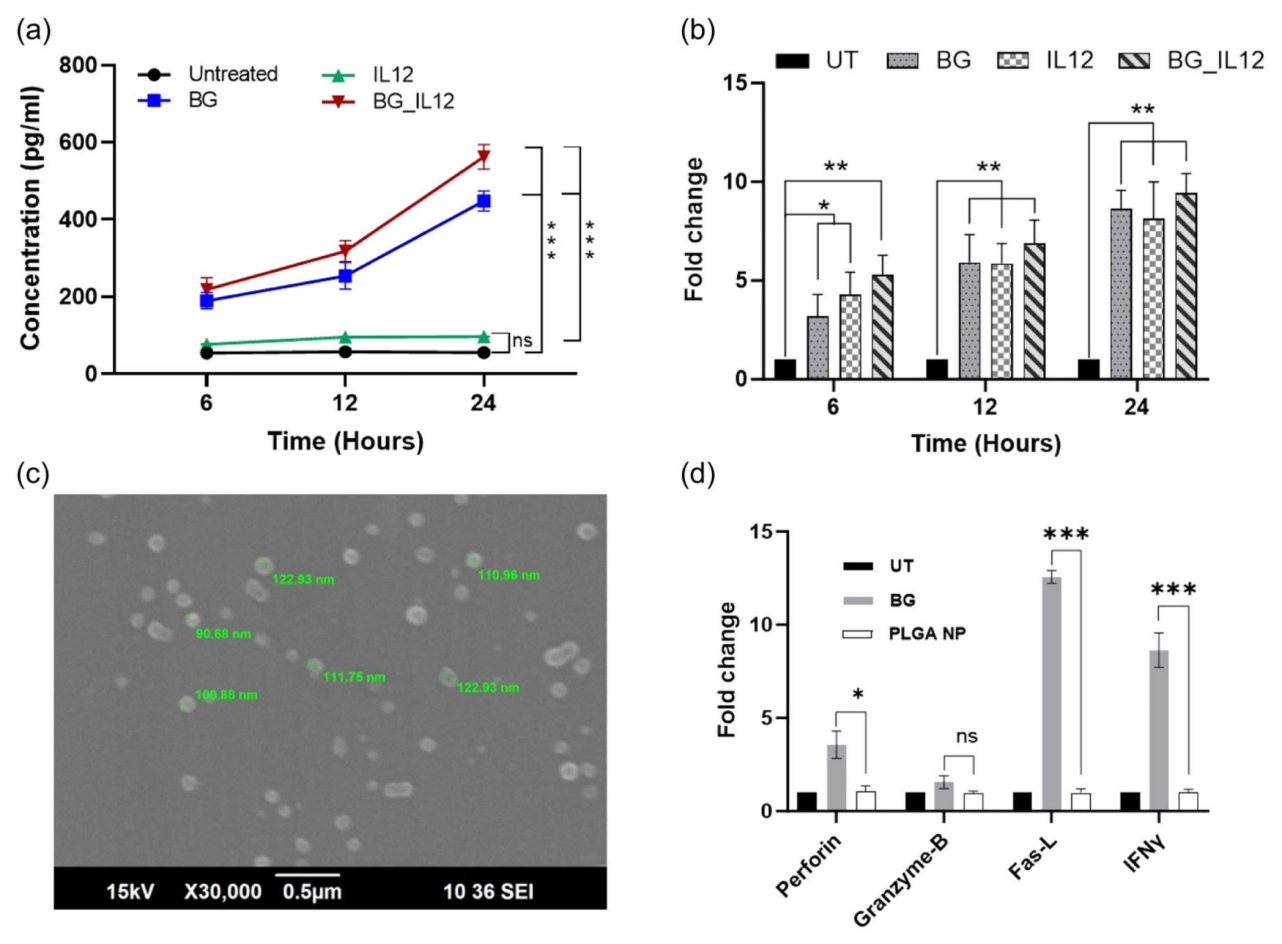
Protein quantification and gene expression analyses. (a) Quantification of IFN-*γ* protein content in the supernatant of BG, IL12, and BG_IL12-treated NK-92 cells at specific time points using a sandwich enzyme-linked immunosorbent assay (ELISA). (b) Relative fold change of IFN-*γ* gene expression estimated using qPCR via the 2^–ΔΔCt^ method for BG, IL12, and BG_IL12-treated NK-92 cells compared to the untreated control, with GAPDH as reference. (c) Scanning electron micrograph of PLGA nanoparticles prepared via nanoprecipitation. (d) Relative fold change of gene expression in NK-92 cells treated with BGs and PLGA nanoparticles performed using the 2^–ΔΔCt^ method via qPCR for perforin, granzyme-B, Fas-L and IFN*γ*, compared to the untreated control, with GAPDH as reference. *p < 0.05, **p < 0.005, ***p < 0.001 and ns: not significant. BG, bacterial ghost; GAPDH, glyceraldehyde-3-phosphate dehydrogenase; IFN, interferon; NK, natural killer.

We were also keen to understand if the BG_IL12 particles could induce the secretion of IFN-*γ* in NK-92 cells. Production of IFN-*γ* by NK cells has been previously reported to be induced synergistically by IL-12, IL-15, and IL-18 combinations (Lusty et al., 2017; Mirjacic Martinović et al., 2015). The induction of IFN-*γ* is perceived as advantageous in the immuno-compromised tumor microenvironment, given its capacity to reverse the immunosuppressive and protumoral properties, and prevent the generation of protumorigenic M2 macrophages (Duluc et al., 2009). We first quantified the content of IFN-*γ* in the cell-free supernatant of the treated cells and found striking disparity in the contents induced by BG and IL12 individual treatments (Figure 5a). The BG treatment had yielded several fold higher quantities of IFN-*γ* (*p* < 0.001) than that induced by IL12 alone. Additionally, the BG_IL12 particle showed a similar trend of IFN-*γ* content as that of the BG treatment. At this point, we decided to revalidate the status of IFN-*γ* by checking the gene expression levels. On analysing the qPCR data, we interestingly observed an obvious nested pattern of time-dependent increase in gene expression for IFN-*γ* in all the three treatments (Figure 5b). As depicted in the figure, all the samples showed significant upregulation in gene expression when compared to the respective untreated controls in a time-dependent manner.

This experiment clearly revealed that though IL12 was able to induce an increase in IFN-*γ* gene expression, it was unable to individually drive the IFN-*γ* secretion in the protein form. Previously, it was reported that IL12 causes IFN-*γ* mRNA accumulation in NK92 cells and requires stimulation with IL-2 (Hodge et al., 2002) or IL-18 (Chaix et al., 2008) to improve its translation. In line with this suggestion, we believe that the BGs have suitably synergized the IFN-*γ* secretion which was otherwise not possible by IL12 alone. This result can be regarded as important in terms of the benefits that can be harnessed by using BGs as carriers/synergizers for cytokines, which otherwise may not be achievable with conventional carrier systems like PLGA nanoparticles. To validate this idea, we prepared PLGA nanoparticles (Figure 5c) via nanoprecipitation method (Supporting information) and compared their NK cell-priming effect with that of BGs. Our data (Figure 5d) indicated that PLGA nanoparticles at the equivalent dose of BG (i.e., 488.28 ng/mL) did not show any influence on the expression of perforin, granzyme-B, Fas-L or IFN-*γ* in NK-92 cells, underscoring the distinct immunostimulatory prowess of BGs and the potential of having them as carriers or adjuvants for cytokine-based NK cell potentiation.

**Figure 6:**
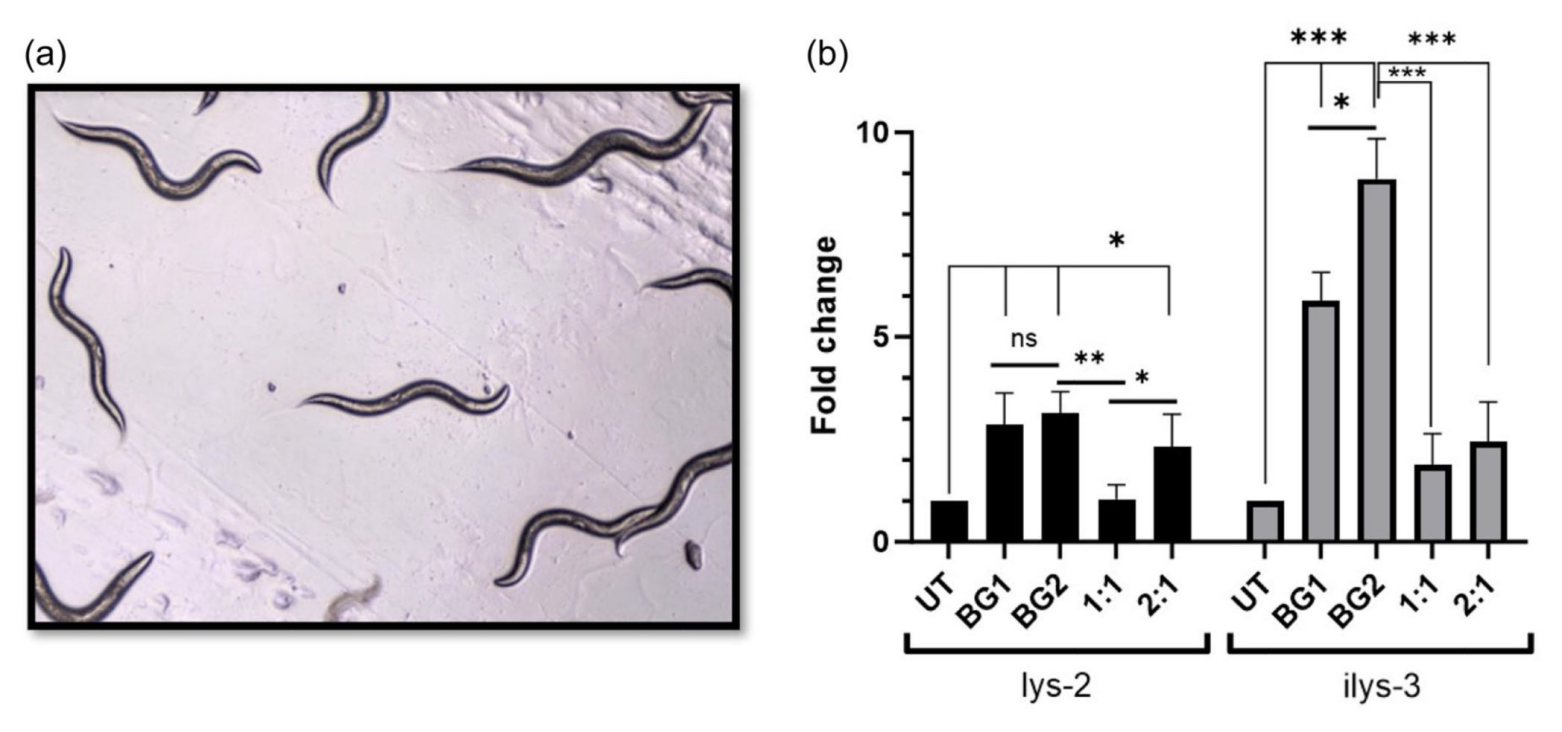
Gene expression analyses in *C. elegans*. (a) Micrographic image of *C. elegans* on Day 0 of treatment. (b) Relative fold change of lys-2 and ilys-3 gene expression estimated via qPCR using the 2^–ΔΔCt^ method and compared with the untreated control, with snb-1 as the reference gene for normalization. The BG:OP50 ratios tested were 1:1 and 2:1 where, 1:1 is 1 × 10^4^:1 × 10^4^ units/plate, and 2:1 is 2 × 10^4^:1 × 10^4^ units/plate of BG:OP50; BG1 = 1 × 10^4^ units/plate and BG2 = 2 × 10^4^ units/plate. *p < 0.05, **p < 0.005, ***p < 0.001 and ns: not significant. BG, bacterial ghost.

Furthermore, we investigated the activation of innate immunity by BGs in the context of a 3D environment. For this, we chose to conduct initial studies on a basic, but robust in vivo model, the *C. elegans* (Figure 6a). *C. elegans* is a nematode widely employed as a versatile animal model in research involving aging, neurodegenerative conditions, and more importantly the innate immune system (Marsh & May, 2012; Shen et al., 2018). They are largely advantageous over other in vivo models as they are relatively inexpensive, have a short generation time, easy to culture and have a completely sequenced genome. In *C. elegans* the innate immune system is activated via highly conserved MAPK signaling pathways and the antimicrobial effector lysozymes are responsible for the activation of innate immunity (Ermolaeva & Schumacher, 2014). Fifteen phylogenetically varied lysozyme genes are present in *C. elegans* (Schulenburg & Boehnisch, 2008), of which, we choose to evaluate two lysozyme genes viz., lys-2 and ilys-3. These genes are normally upregulated during bacterial infection as a defense mechanism against gram-positive bacteria (Boehnisch et al., 2011). ilys-3 is involved in the degradation of endocytic cargo, such as proteins, lipids and carbohydrates, while lys-2 is implicated in the degradation of autophagosomes, and both genes are regulated by the ERK-MAPK-pathway (Gravato-Nobre et al., 2016). Since the BGs were derived from gram-positive bacteria and because the BGs are protein-lipid-carbohydrate structures, we particularly chose the above-mentioned genes for evaluation.

For the qPCR study we created two scenarios: one, in which different concentration of BGs were used (BG1: 1 × 10^4^ units/plate, BG2: 2 × 10^4^ units/plate) and, second, in which combinations of BG and OP50 (nematode food) were used. As depicted in Figure 6b, we first observed that both the gene viz., lys-2 and ilys-3 were influenced by the ghost cell treatment and showed upregulation in gene expression though at differing folds. But interestingly, in the case of lys-2, though BGs could upregulate the gene expression significantly at 1 × 10^4^ units (BG1), we observed that doubling the BG dosage (BG2) did not have any significant influence on the gene expression fold change. However, in contrast, there was significant gene upregulation of ilys-3 by the BGs in a dose-dependent manner.

In the second scenario, we combined different dosages of BGs (BG1 and BG2) with a single dose of OP50. We found that 1:1 ratio of BG:OP50 did not have any influence in the fold change of lys-2 gene expression, while the expression increased when the BG dosage was enhanced. This indicated that *C. elegans* has a certain preference for its usual food OP50 when available in plentiful. However, the worm would shift its preference to BGs when the BG dosage is doubled and is encountered more frequently than OP50, during the feeding time. In the case of ilys-3 we also observed an increase in gene expression after BG treatment (as discussed earlier), but noteworthily, the expression was significantly lower (*p* < 0.005) for the combinations of BG with OP50, repeatedly indicating a strong preference for OP50 even when the BG dosage was doubled. Taking our results together, we understand that the BGs by themselves could influence the two important innate immunity genes in *C. elegans* in a positive way. However, we suggest that future immunomodulatory investigations on *C. elegans* should be performed in the absence of OP50, to understand the scenario in a more controlled and uninterrupted manner.

## 4 Conclusion

In the realm of cancer treatment, immunotherapy has emerged as a preferred approach, and NK cells have garnered significant attention for their cytotoxicity and ability to produce immunoregulatory cytokines. Our study focuses on developing cost-effective modalities for NK cell-based immunotherapy, with a particular emphasis on utilizing BGs as potential candidates. These offer several advantages such as easy production, cost-effectiveness, and the inherent presence of immunomodulatory biomolecules. Our findings highlight the efficacy of BGs as potentiators of NK cells and their capacity as supportive adjuvants for interleukin-12, a therapeutic agent with limited success in clinical trials. Additionally, we demonstrate that BGs can activate key innate immunity genes in *C. elegans*, extending our *proof*-*of*-*concept* to an in vivo setting. By shedding light on the remarkable therapeutic potential of BGs, our study serves as a catalyst for incisive investigations that delve deeper into harnessing their multifaceted capabilities, not only in addressing the exigencies of cancer treatment but also in combating infectious diseases and advancing prophylactic interventions.

## Supplementary Material

Supplementary Material

## Figures and Tables

**Table 1:** List of forward and reverse primers for the investigated genes.

Gene	Forward primer sequence	Reverse primer sequence
Perforin (PRF-1)	5′-CGCCTACCTCAGGCTTATCTC-3′	5′-CCTCGACAGTCAGGCAGTC-3′
Granzyme B (GZMB)	5′-TGGGGGACCCAGAGATTAAAA-3′	5′-TTTCGTCCATAGGAGACAATGC-3′
Fas Ligand (FasL)	5′-TCAATGAAACTGGGCTGTACTTT-3′	5′-AGAGTTCCTCATGTAGACCTTGT-3′
Interferon-gamma (IFNG)	5′-TGACCAGAGCATCCAAAAGA-3′	5′-CTCTTCGACCTCGAAACAGC-3′
Glyceraldehyde-3-phosphate dehydrogenase (GAPDH)	5′-CGACCACTTTGTCAAGCTCA-3′	5′-AGGGGAGATTCAGTGTGGTG-3′

**Table 2:** List of forward and reverse primers for the investigated genes in *C. elegans*.

Gene	Forward primer sequence	Reverse primer sequence
Lysozyme-like protein-2 (lys-2)	5′-GACGTTGGCAGTTGGATTG-3′	5′-GCTGGATTGGGAATTGAGAC-3′
Invertebrate Lysozyme-3 (ilys-3)	5′-ATGCCAAAATCAAATGCACA-3′	5′-CCACCTAAACACTTCCGTCC-3′
Synaptic vesicle protein (snb-1)	5′-CCGGATAAGACCATCTTGACG-3′	5′-GACGACTTCATCAACCTGAGC-3′

## Data Availability

The data that support the findings of this study are available from the corresponding author upon reasonable request.
