# Supplementary material for: Demonstrating the immunostimulatory and cytokine-augmentation effects of bacterial ghosts on natural killer cells and Caenorhabditis Elegans
Source: Biotechnol Bioeng. Author manuscript; Available in PMC 2024 Mar 23. (PMC7615764; doi:10.1002/bit.28619)
Supplement: Supplementary Material [file EMS194834-supplement-Supplementary_Material.docx]

**Preparation, characterisation of PLGA nanoparticle**

PLGA nanoparticles were prepared through the nanoprecipitation route. 25 mg PLGA (50:50, M_H_:  22- 45K, Polysciences) was dissolved in an organic phase consisting of a non-solvent (5 ml chloroform, Sigma) at 1: 10 v/v ratio. The organic solution was then added drop wise to 10 ml of distilled water containing 0.5% *w*/*v* of Pluronic F-68 (Sigma) and stirred overnight to remove solvent. Nanoparticles were then recovered from the nanodispersion by centrifugation for 30 min at 25000 rpm, and washed two times with distilled water. The nanoparticles were size characterised using scanning electron microscope (JSM 6490 LA, JOEL, Japan).

**Cytotoxicity**

To test the cytotoxicity, NK-92 cells at seeding density of 3.5 x 10^5^ cells/well were incubated with differing concentrations of testing samples (0 – 2.5 μg/ml) in serum-free culture medium for 24 hrs. The cell viability was determined using MTT (3-(4,5-dimethylthiazol-2-yl)-2,5-diphenyl tetrazolium bromide) assay. Briefly, the treated cultures were centrifuged at 400 x g to remove the supernatant and the cell pellet was subjected to 100 μl of 0.25 mg/ml MTT-tetrazolium salts (Sigma-Aldrich, USA) in PBS followed by incubation for 4 hrs at 37°C. The formazan crystals were solubilized using DMSO and the absorbance was measured using a Tecan spectrophotometer at a wavelength of 570 nm. The experiments were performed in three independent sets of triplicates and the cell viability was determined in percentage values.


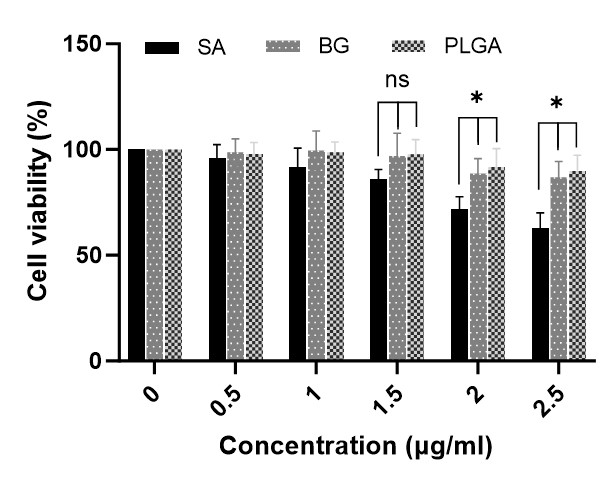


Figure S1: Cytotoxicity evaluation by MTT assay on NK-92 cells treated with BG, live S. aureus (SA) and PLGA nanoparticles. The graph illustrates the relative cell viability percentages calculated with 0 µg/ml BG/SA/PLGA (ie. Medium alone) as control. *p-value<0.05, **p-value<0.005, ns: not significant.
